# Recovering *Cucurbita pepo* cv. ‘Lungo Fiorentino’ Wastes: UHPLC-HRMS/MS Metabolic Profile, the Basis for Establishing Their Nutra- and Cosmeceutical Valorisation

**DOI:** 10.3390/molecules24081479

**Published:** 2019-04-15

**Authors:** Simona Piccolella, Alessandro Bianco, Giuseppina Crescente, Alessandra Santillo, Gabriella Chieffi Baccari, Severina Pacifico

**Affiliations:** Department of Environmental Biological and Pharmaceutical Sciences and Technologies, University of Campania “Luigi Vanvitelli”, Via Vivaldi 43, I-81100 Caserta, Italy; simona.piccolella@unicampania.it (S.P.); alessandro.bianco55@gmail.com (A.B.); giuseppina.crescente@unicampania.it (G.C.); alessandra.santillo@unicampania.it (A.S.); gabriella.chieffi@unicampania.it (G.C.B.)

**Keywords:** *Cucurbita pepo* cv. ‘Lungo Fiorentino’, food waste, UHPLC-HRMS, polyphenols, cosmeceutical valorisation

## Abstract

Food-waste is produced throughout all the food supply chain, with a large part already achieved at farm level. In fact, fruits and vegetables, which do not satisfy aesthetic demands, cannot be marketed, but their recovery could favour their valorisation for the obtainment of highly qualified goods. In this context, faulty zucchini fruits (cultivar ‘Lungo Fiorentino’), intended for disposal, were rescued as effective, inexpensive and bio-sustainable source for cosmeceutical purposes. Zucchini fruits underwent extraction and fractionation to obtain ZLF-O and ZLF-A extracts, which were chemically characterized by UHPLC-HRMS. ZLF-A extract, rich in flavonols and flavones, scavenged massively DPPH^•^ and ABTS^•+^, and was not cytotoxic at doses up to 200 μg/mL. Thus, ZLF-A was incorporated into a base cream formula. Zucchini-based emulsion was deeply screened for its antiradical properties and cytotoxicity towards human keratinocytes and fibroblasts. ZLF-A-enriched cream, whose chemical stability was assessed over time and mimicking different storage conditions, was further tested on reconstructed epidermis disks (Episkin^TM^). The recovery of valuable chemical substances from zucchini agro-food waste, complying with the principles of valorisation and sustainable development, can represent a new market force for local farmers. Data acquired were eager to convey a suitable reuse of nutraceuticals rich zucchini waste.

## 1. Introduction

A large amount of waste is produced along the entire agri-food supply chain, causing considerable concern for environmental sustainability, waste of resources and human health. Indeed, it is broadly demonstrated that agri-food by-products and wastes are rich in valuable bioactive compounds, whose valorisation could reach targeted applications in diverse biotechnological fields: from the pharmaceutical sector to the food industry, up to the rapidly evolving cosmetics sector [1]. This latter carefully requires innovative and functional natural compounds and extracts, as a clear response to consumers’ concerns about synthetic substances [2]. In this context, polyphenols, thanks to their anti-oxidant and anti-inflammatory activities [3], as well as their anti-photocarcinogenic effects [4], are attractive ingredients for cosmetics [5], and plant oils are considered beneficial in wound healing promotion and skin barrier repair [6]. Natural products recovery, according to sustainable waste and resource management principles, from agri-food wastes, non-responding to specific requests (of appearance, shape, colour and size) of the large-scale agri-food market, represents an effective, inexpensive and bio-sustainable nutraceutical source [7,8]. Olive by-products (e.g., leaves, stones, mill waste water, mill pomace), for example, extremely rich in low-molecular-weight antioxidant phenols, and fatty acids, were hypothesised, based on their anti-inflammatory, anti-atherogenic, antitumor, antimicrobial, antiviral activities [9], to be a source of active ingredients for cosmetics with different claims, such as anti-aging or hydration [10]. The phenols recovery from olive mill wastewater was recently experimented for application as UV booster in cosmetics [11]. *Citrus* processing also generates a high amount of agricultural waste. A survey carried out on *Citrus* × *unshiu* ethanol extracts, highlighted their positive effects on some atopic dermatitis markers [12], and quercetagetin was found to efficaciously inhibit TARC and MDC expression in IFN-γ and TNF-α stimulated HaCaT human keratinocytes [13]. Winery wastes and by-products, both liquid and solid, as part of the sustainability drive, were increasingly under the microscope for their high content in phenols and polyphenols, whose incorporation into functional foods, nutraceuticals, and cosmetics, could benefit humans in preventing multiple degenerative conditions [14]. Moreover, coffee processing generates large amounts of unused beans, because they are mechanically damaged or not sufficiently ripe or developed to move on to the next processing phase. The recovery of phenol antioxidants in unused coffee beans through the production of their ethanol extract was showed to improve skin renewal and to strengthen the epidermal barrier, preventing water loss, inhibiting melanin synthesis and accelerating repair of damaged epidermal cells [15]. Thus, agri-food wastes captivating composition, combined with sustainability issues, make the recovery of target compounds from wastes an advantageous opportunity, from which cosmetic field may particularly benefit for skin health preservation. 

It is worth of note that factors, such as the crop type, the economic development levels, and, mainly, the non-compliance with the quality standards set by retailers, could strongly influence agri-food loss in the initial production stages (at farm level) [16,17]. In this case, the food, even if produced, is not able to be included in the supply chain, but its recovery needs to be pursued for reducing its environmental impact, and disposal costs, and more importantly, for converting this waste, which is still fit for human consumption, in highly qualified products. 

In this context, the recovery and cosmeceutical valorisation of zucchini (cultivar ‘Lungo Fiorentino’), with no suitable requirements for sale, was investigated. Zucchini ‘Lungo Fiorentino’ have long, cylindrical, ribbed, green fruits with lighter ribs. The fruit, appreciated for the flavour and the tender consistency of the pulp, is picked up still small (15–20 cm), with the characteristic flower. The waste material consisted in faulty zucchini fruits, out of gauge, damaged by bad weather, badly grown, without the aesthetic marketing features. Ugly zucchini underwent extraction and fractionation preparing extracts with distinct polarity. These latter were chemically characterized in their bioactive constituents by ultra-high-pressure liquid chromatography-high resolution tandem mass spectrometry (UHPLC-HRMS) techniques. In order to highlight a cosmeceutical potential of extracts obtained, the same, in the different phases of fractionation, were subjected to bioactivity tests for the assessment of the antiradical properties. Cytotoxicity evaluation was also carried out in order to incorporate safe bioactive extracts into a base cream formula. Zucchini-based cream, as well as zucchini extracts were tested on reconstructed epidermis disks (EpiSkin, Recostructed Human Epidermis, small, age day 17 0.50 cm^2^).

## 2. Results and Discussion

### 2.1. Metabolic Profiling of ZLF-A Fraction

The UHPLC-HRMS investigation of ZLF-A from XAD-4 fractionation led to the tentative identification of eighteen metabolites, fifteen of which were flavonoid glycosides differing for the aglycone and the number/type of saccharidic moiety. 

In Table 1 UHPLC-HRMS and MS/MS data for all compounds were summarized, together with theoretical *m*/*z* values, calculated molecular formulas, errors (ppm) and the tentative assignment.

Compound **1** was tentatively identified as the *O*-hexoside of the flavonol myricetin. In fact, the deprotonated pseudomolecular ion at *m*/*z* 479.0826 was in accordance with the molecular formula C_21_H_20_O_13_ (−1.0 ppm error). The neutral loss of 162/163 Da, typical of hexoses O-linked to an aglycone, led to the product ions at *m*/*z* 317.0313 and 316.0218, whose intensity ratio suggested 3-*O*-glycosylation [18].

The [M−H]^−^ ion detected for compound **2** at *m*/*z* 755.2025 (−2.0 ppm error) suggested the molecular formula C_33_H_40_O_20_ and a ‘Rings and Double Bonds’ (RDB) value equal to 14. Following collision induced dissociation, it gave rise to the main product ions at *m*/*z* 301.0355/300.0277, putatively ascribed to the flavonol quercetin. Thus, a neutral loss of 454/455 Da occurred, which was supposed to be the glyconic moiety of the molecule, likely a trisaccharide formed by one hexose and two deoxyhexoses. The presence in the TOF-MS^2^ spectrum of very low fragment ions let us to hypothesize the connection among sugars (see supplementary materials, Figure S1). In fact, the first one at *m*/*z* 609.1484 ([M−H−146]^−^, loss of one deoxyhexose moiety), once formed, generated the ion at *m*/*z* 489.1059 through the loss of 120 Da, which resembles a cross-ring cleavage of hexose sugars. This evidence could indicate a connection between the remaining sugars which involves hexose C2 position and likely the anomeric deoxyhexose moiety [18].

In addition, compounds **3**, **5** and **6** were also putatively characterized as quercetin glycosides. The deprotonated aglycone ions were formed in these cases following neutral losses likely attributable to a hexosylpentose (294 Da, e.g., sambubiose), a hexosyldeoxyhexose (308 Da) and a hexose (162 Da), respectively. The identity of metabolite **5**, detected as the main constituent of ZLF-A fraction (Table S1), was further confirmed by comparison with data recorded for the pure commercial rutin.

Compounds **4**, **10**, **11**, **13** and **14** shared the same deprotonated aglycone ions, likely attributable to the flavonol kaempferol, based on the occurrence of the peak at *m*/*z* 285.039 and other characteristic product ions at *m*/*z* 284.032 (deprotonated aglycone ions, deriving from the homolytic cleavage of the saccharidic unit), 255.03 and 227.03. Metabolite **4**, with a molecular formula C_33_H_40_O_19_ and RDB value equal to 14, could be identified as clitorin, a glycoside bearing a trisaccharide formed by one glucose and two rhamnose residues linked to C-2″ and C-6″ positions. This hypothesis found confirmation in the TOF-MS^2^ spectrum, where the first product ion at *m*/*z* 575.1429 was generated by the neutral loss of 164 Da, likely a deoxyhexose residue, which in turn fragmented to give the deprotonated kaempferol ([M−H−164−290 Da]^−^). To the best of our knowledge, this glycoside has never been described in C. pepo. Furthermore, the putative presence of the isomer robinin, already reported as constituent of zucchini by Iswaldi et al. [19], could be excluded. In fact, in this latter molecule one rhamnose unit is bound directly to kaempferol at C-7 position (aromatic ring A). Thus, as previously reported [20], the collision-induced dissociation (CID) would have provided the product ion at *m*/*z* 593 ([M−H−146 Da]^−^, Y_7_^−^), not detected in our experiments.

TOF-MS^2^ mass spectra of compounds **10** and **13** at *m*/*z* 593.1514 (or 593.1520) were in accordance with the presence of two O-hexosyldeoxyhexosyl derivatives of kaempferol, differing in the sugar position and/or in the interglycosidic linkage [21]. They have been already tentatively identified in zucchini [19]. Instead, [M−H]^−^ ion for compound **11** at *m*/*z* 579.1358 was in accordance with a molecular formula C_26_H_28_O_15_ (0.4 ppm error; RDB value of 13). Its fragmentation pattern highlighted the presence of a hexosylpentoside moiety bound to kaempferol at C3 position. Indeed, recently Abu-Reidah et al. [22] described kaempferol 3-sambubioside as a polyphenolic constituent of cucumber whole fruit extract. Finally, deprotonated compound **14** at *m*/*z* 447.0924 (−2.4 ppm error) with a molecular formula equal to C_21_H_20_O_11_ and RDB value of 12 was tentatively identified as kaempferol 3-O-hexoside, based on its fragmentation pattern previously reported in literature [23].

TOF-MS^2^ spectrum of deprotonated compound **7** at *m*/*z* 769.2183 showed the same neutral losses of 164 and 290 Da observed for metabolite **4**, allowing us to hypothesize the occurrence of the same glycosidic moiety, linked in this case to a different aglycone (*m*/*z* 315.0514/314.0439), putatively identified as isorhamnetin (methylquercetin). The presence of the ion at *m*/*z* 299.0204, likely formed through the loss of a methyl radical from the aglycone ion, seemed to confirm our hypothesis. Metabolites **12** and **15** were also putatively identified as isorhamnetin glycosides. They were isomers, showing the same molecular formula equal to C_28_H_32_O_16_. In particular, from deprotonated pseudomolecular ions at *m*/*z* 623.1619 (or 623.1620) neutral losses of 308.1147 (or 308.1148) Da occurred to generate the aglycon ions, thus suggesting the presence of a hexosyldeoxyhexose (e.g., rutinose) moiety linked. A different glycosylation position was supposed, based on the different intensity ratio of Y_0_^−^ and [Y_0_−H]^−^ ions [24].

Compounds **8** and **9** could be *O*-glycosides of the flavone luteolin. In fact, in the collision induced dissociation process from deprotonated precursor ions at *m*/*z* 447.0925 and 593.1506 the same aglycone ion was generated at *m*/*z* 285.0395 (or 285.0404). The TOF-MS^2^ spectra differed from those recorded for kaempferol derivatives for the absence of fragments typical of flavonols, such as *m*/*z* 271.0246, 255.0302, 227.0342. Based on neutral losses of 162 and 308 (162 + 146) Da, which led to the aglycone ions, these compounds were putatively identified as luteolin *O*-hexoside and luteolin *O*-hexosyl-deoxyhexoside, respectively. The occurrence of luteolin and its *O*-glycosides has been already reported in literature in zucchini, as well as in other plants belonging to Cucurbitaceae family [19,25].

The molecular formulas calculated for metabolite **16** and **17**, based on deprotonated ions at *m*/*z* 715.1893 and 699.1942, were C_34_H_36_O_17_ and C_34_H_36_O_16_, respectively. The tentative chemical characterization of these molecules was based on their fragmentation patterns, which showed the same neutral losses, thus generating product ions, each differing for 16 Da. In Figure S2 the TOF-MS^2^ spectra are reported, together with the hypothesized fragmentation pathways. Briefly, precursor ions underwent the loss of a phenol moiety to give the ions at *m*/*z* 621.1469 (or 605.1517). The subsequent loss of 308.1130/309.1206 Da could be indicative of the presence of a disaccharide (hexose + deoxyhexose), as commonly observed for other polyphenols. The molecular formulas of [aglycone-H]^−^ ions at *m*/*z* 313.0346 and 297.0385 were C_16_H_9_O_7_^−^ and C_16_H_9_O_6_^−^ and could be described as acetylated anthraquinones differing for one hydroxyl group. Confirming the presence of an acetyl group, lower fragments deriving from the neutral loss of 58 Da, as the ion at *m*/*z* 333.0745 in the TOF-MS^2^ spectrum of compound **17**, were detected. To the best of our knowledge glycosylated or acetylated anthraquinone derivatives have never been identified in zucchini, but they are quite common in other plants, such as Cassia species [26,27,28].

Metabolite **18** showed a fragmentation pattern, which resembles the one observed for ent-kaurene diterpene glycosides (e.g., steviol glycosides) previously detected in Stevia rebaudiana leaves [29]. In fact, the neutral loss of two hexose moieties from the deprotonated pseudomolecular ion at *m*/*z* 803.3713 (C_38_H_60_O_18_, 0.8 ppm error) gave rise to the ion at *m*/*z* 479.2673, which in turn underwent a further deglycosylation to produce the ion at *m*/*z* 317.2129. This latter was putatively identified as hydroxykaurenoic acid (e.g., steviol), in accordance with a molecular formula C_20_H_30_O_3_ with RDB value equal to 6. To confirm this hypothesis, TOF-MS and MS^2^ spectra of compound **18** and of rebaudioside A commercial standard were compared (Figure S3). It is worth of note that kaurenoic acid is involved in the biosynthesis of several plant growth regulators, thus its derivatives can be mostly found in different plant species belonging to several families [30]. The occurrence in C. pepo of hydroxykaurenoic acid derivatives has been previously reported in literature [31].

### 2.2. Metabolic Profiling of ZLF-O Fraction

**Figure 1:**
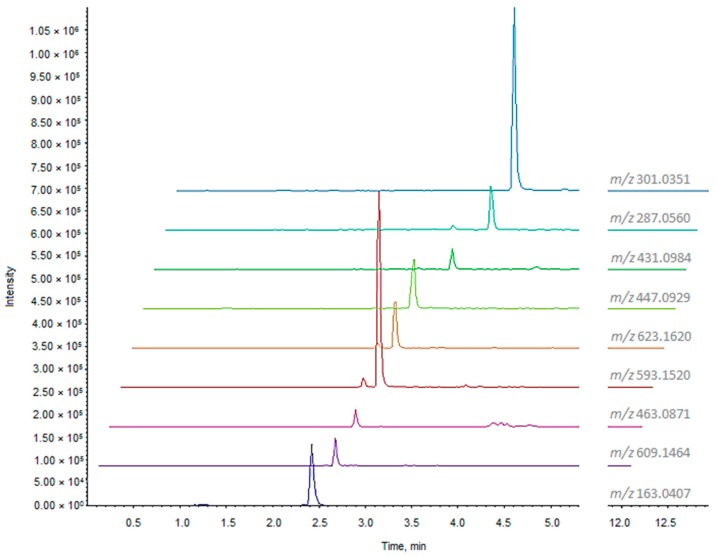
XIC chromatograms of phenols and polyphenols tentatively identified in ZLF-O fraction.

The UHPLC-HRMS analysis of ZLF-O extract (Table 2) revealed the presence at lower retention times of glycosylated flavonoids, some of which previously described as components of the ZLF-A fraction, such as quercetin hexoside (*m*/*z* 463.0871; C_21_H_20_O_12_) and rutin (*m*/*z* 609.1464; C_27_H_30_O_16_), kaempferol and isorhamnetin rutinoside (*m*/*z* 593.1520, C_27_H_30_O_15_ and *m*/*z* 623.1620, C_28_H_32_O_16_, respectively). Extracted ion chromatograms (XICs) of phenols and polyphenols are reported in Figure 1.

For the metabolite eluting at t_R_ = 3.53 min the molecular formula C_15_H_12_O_6_ was calculated (−0.4 ppm error). In this case, the high-resolution MS/MS spectrum allowed us to distinguish between three possible isomers that differ in the position of a hydroxyl group: flavanons eriodictyol and cartamidin (6-hydroxynaringenin), or favanonol aromadendrin (or dihydrokaempferol). Product ions at *m*/*z* 151.0027 (C_7_H_3_O_4_^−^) and 135.0449 (C_8_H_7_O_2_^−^), formed during the ring C fragmentation, were pivotal in the choice of cartamidin as the most plausible one.

The only phenol, detected at *m*/*z* 163.0407, has been putatively identified as p-coumaric acid, based on the molecular formula C_9_H_8_O_3_ and the decarboxylation of the precursor ion, leading to the fragment at *m*/*z* 119.0508 ([M−H−44 Da]^−^) (Table 2).

**Figure 2:**
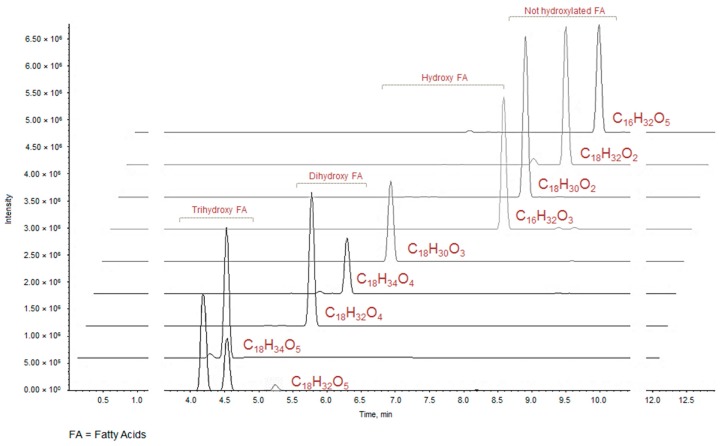
XIC chromatograms of fatty acids tentatively identified in ZLF-O fraction.

At higher retention times (>3.63 min) metabolites detected in the UHPLC-MS analyses could be C18 fatty acids differentiated by the unsaturation degree, except for compounds **23** and **26**, which are constituted by 16 carbon atoms and could correspond to the hydroxypalmitic and palmitic acid, respectively [32]. The elution order reflects the polarity conferred by the presence/absence and by the number of OH groups on the carbon chain. Therefore, it is not surprising that the first eluted are trihydroxyoctadecadienoic and trihydroxyoctadecenoic acids (C_18_H_32_O_5_, *m*/*z* calc. 327.2177 and C_18_H_34_O_5_, *m*/*z* calc. 329.2335, respectively) (Table 2, Figure 2).

### 2.3. Antioxidant Capacity

**Figure 3:**
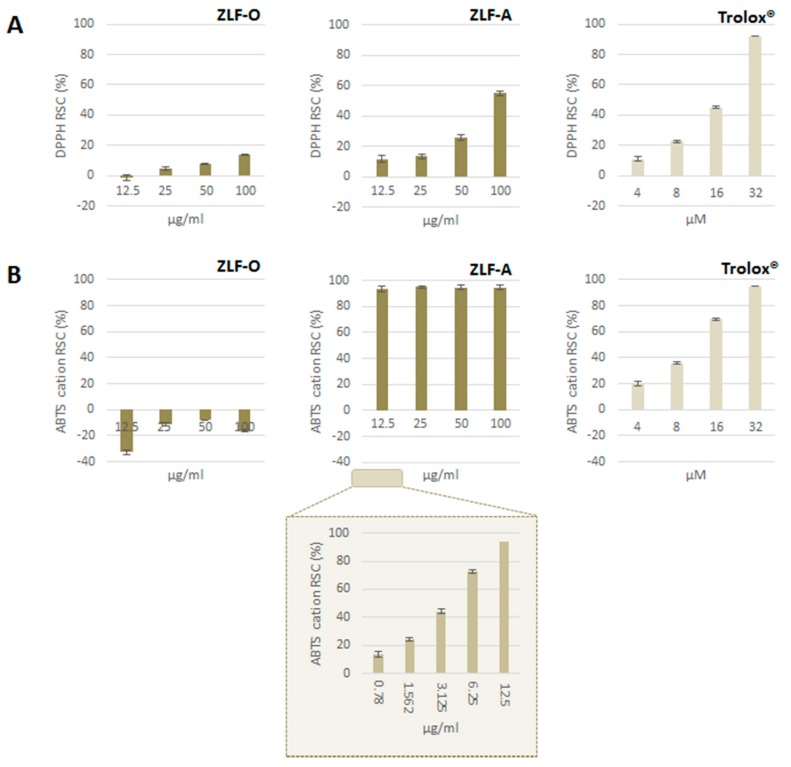
(**A**) DPPH^•^ and (**B**) ABTS^●+^ Radical Scavenging Capacity (RSC, %). Values, reported as percentage vs. blank, are the mean ± SD of measurements carried out on 3 samples (n = 3) analyzed three times.

Extracts obtained from ZLF fractionation underwent Folin-Ciocalteu assay to estimate their relative total phenol content. This latter was expressed as gallic acid equivalents (GAE) per g of extract: ZLF-A was the fraction most enriched in (poly)phenols (118.6 ± 1.6 mg GAE g^−1^), followed by ZLF-O (109.2 ± 1.9 mg GAE g^−1^). ZLF-W1, deriving from the fractionation of the initial aqueous extract, contained the lowest amount of (poly)phenols (16.0 ± 2.0 mg GAE g^−1^). Furthermore, DPPH^·^ and ABTS^+^ methods were applied. ZLF parental extract, ZLF-W, and the derived ZLF-W1 proved to be ineffective as DPPH radical scavengers (data not shown). Similarly, ZLF-O did not show antiradical capacity (Figure 3A), whereas ZLF-A exerted a dose-dependent radical scavenging capacity, with an ID_50_ value equal to 92.8 µg/mL. The obtained results were also evaluated with reference to a positive standard (Trolox^®^), and the TEAC (Trolox^®^ Equivalents Antioxidant Capacity, μg Trolox^®^ per g of extract) value of 7.13 was calculated for ZLF-A.

Analogously, ZLF-O was not able to efficaciously scavenge ABTS^●+^, while ZLF-A revealed an extremely high activity, even at the lowest tested dose (12.5 µg/mL) (Figure 3B). The observed RSC (%) values showed the need to further dilute the sample up to 0.78 µg/mL, thus highlighting that its massive antiradical potential was strongly dose-dependent in the concentration range 0.7–12.5 µg/mL. An ID_50_ value of 3.68 µg/mL was calculated, and the TEAC value was equal to 0.40 µg/mL. ZLF-O and ZLF-A chemical composition could explain their difference in antiradical capacity. In fact, ZLF-A, mainly enriched in flavonoids, was more likely to elicit radical trapping effects.

**Figure 4:**
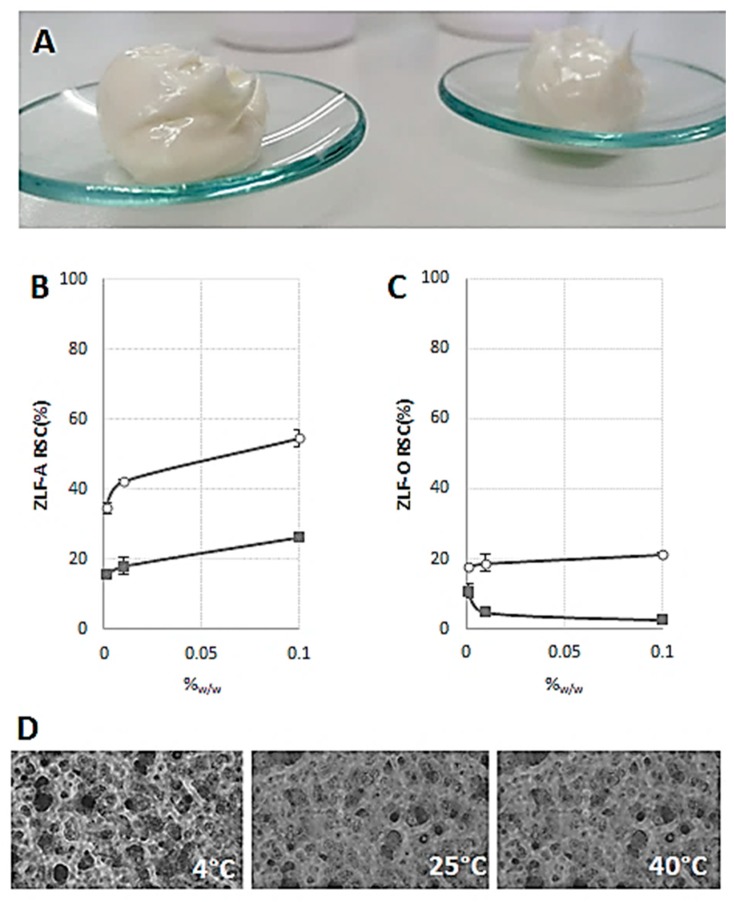
ZLF-A enriched emulsion (**A**); Radical Scavenging Capacity (RSC, %) of ZLF-A enriched emulsion (**B**) and ZLF-O enriched emulsion (**C**) towards DPPH radical (■) and ABTS radical cation (○). Values, reported as percentage vs. a blank, are the mean ± SD. Representative microscopy images of ZLF-A enriched emulsion stored at 4 °C, 25 °C and 40 °C (**D**). Images were acquired by Nikon Eclipse TE300 microscope.

The relative addition of both ZLF-O and ZLF-A extracts in cosmetic emulsion (Figure 4A) highlighted ZLF-A antiradical capability. In fact, ZLF-A enriched emulsion, whose optical microscopy images are in Figure 4 (panel D), appeared to be able to scavenge ABTS radical cation, reaching a RSC(%) value equal to 54%, when 0.10%_w/w_ ZLF-A enriched the base emulsion (Figure 4B). The dose-response effect was also highlighted in the DPPH radical scavenging capacity assay, whereas ZLF-O-based cream did not lead to effective response (Figure 4C).

### 2.4. Cytotoxicity Screening

**Figure 5:**
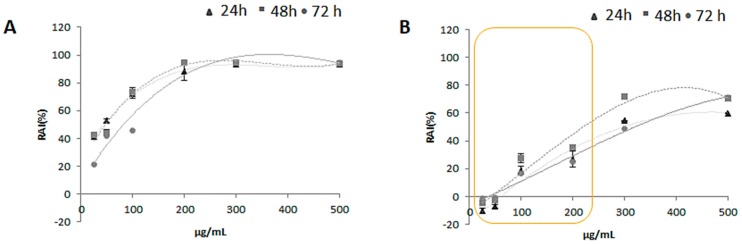
Mitochondrial redox activity inhibition (RAI %) estimated by MTT test in HaCat cell line treated with increasing doses (25.0, 50.0, 100.0, 200.0, 300.0 and 500.0 μg/mL) of (**A**) ZLF-O and (**B**) ZLF-A extracts. Values, reported as percentage vs. an untreated control, represent mean ± standard deviation (SD) of measurements carried out in 3 samples (n = 3) analyzed twelve times.

For the in vitro cytotoxicity screening MTT test was carried out on HaCat human keratinocytes cell line, which represents a valid model in dermoprotection studies. To this purpose cells were treated with increasing doses (25.0, 50.0, 100.0, 200.0, 300.0 and 500.0 μg/mL) of zucchini extracts (ZLF-O and ZLF-A) for three exposure times (24, 48 and 72 h). Both the extracts showed a remarkable antiproliferative potential on the human keratinocytes cell line, with a stronger inhibiting potential on the cellular redox activity exerted by ZLF-O (Figure 5). In fact, for this latter the greatest cytotoxic potential was detected at 48 and 72 h exposure times at the 200.0 μg/mL tested dose. At higher doses for all exposure times the trend of the curves became almost constant. Instead, in the case of ZLF-A when cells were treated with doses of 25.0 and 50.0 μg/mL no inhibitory activity was recorded at the three exposure times. Then, the cytotoxic potential showed a dose-dependent increase, reaching the maximum of 70% of redox activity inhibition (RAI) at the highest tested dose (500.0 μg/mL) after 48 and 72 h.

### 2.5. Cytotoxicity Screening of a Cosmeceutical Formulation Containing ZLF-A Extract

MTT test data on HaCaT cell line put the basis for the usage of ZLF-A extract in the development of a cosmeceutical preparation, which could guarantee a beneficial effect on skin cells, following a constant and prolonged application. To this purpose, the prepared cosmetic emulsions (0.10%_w/w_ of added ZLF-A extract) underwent an in vitro screening, in order to highlight its cytotoxic potential towards HaCat human keratinocytes and MRC-5 human fibroblasts. Cells were exposed for 15′ or 2 h 30′ to ZLF-A enriched emulsion. HaCat cell line proved to be moderately sensitive to the short-term exposure. In fact, it was observed that, after 15 min exposure time, ZLF-A enriched cream inhibited by 26.7 ± 0.8% the mitochondrial redox activity; its effect was slightly higher than that estimated for the base emulsion, used as control (24.5 ± 0.5%). When the exposure time increased, cell adaptation occurrence seemed to be defined as cell viability decrease was calculated equal to 16.7 ± 0.7%.

MRC-5 cell line showed an interesting responsiveness to the MTT assay, in that it revealed a more pronounced resistance to the treatment with the cosmeceutical preparation containing ZLF-A extract. In fact, the initial mitochondrial redox activity inhibition (19.6 ± 0.4%), evaluated at 15 min exposure time, decreased to 12.8 ± 0.7% after 2 h 30 min.

In order to further evaluate the safety of ZLF-A enriched cosmetic emulsion, an in vitro reconstructed human epidermis (RhE; EpiSkin**^™^**), from normal human keratinocytes and therefore histologically similar to the in vivo tissue, was used. Due to the ban of animal experiments taking place in March 2009 (7th amendment of the cosmetic directive, EU 2003), for its ability to mimic the human skin this model represents an effective alternative [33]. After validation by the European Union (EU), it was included in the relevant EU Test Method Regulation (440/2008/EC) [34] and, also, certified by the Organization for Economic Cooperation and Development (OECD) [35]. Following manufacturer recommendations, MTT test was applied after exposure of the RhE to the emulsion containing 0.010 and 0.10%_w/w_ of ZLF-A extract.

**Figure 6:**
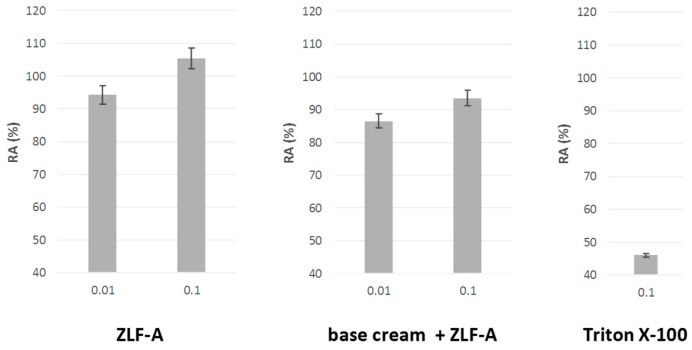
Mitochondrial redox activity (RA (%)) estimated by MTT test in Episkin^™^ reconstructed human epidermis exposed to ZLF-A crude extract and ZLF-A-enriched cosmeceutical formulation.

The mitochondrial redox activity (RA) of the cells constituting the reproduced epidermis was compared to those obtained with the crude extract and with Triton X-100, used as positive standard, able to induce skin irritation (Figure 6).

The results highlighted a very favourable outcome in terms of residual cellular redox activity following application. In fact, the RA% of the emulsion containing the lower extract amount (0.010%_w/w_) was equal to 86.5 ± 0.9% and increased to 93.6 ± 0.8% when the concentration was 10-fold higher. A similar trend was observed after the exposure to the crude plant complex, in that the increase of the extract tested dose resulted in an enhanced RA% (from 94.4 ± 0.6% up to 105.3 ± 0.6%), demonstrating that the treatment ensured cytoprotection, cell proliferation, preservation of mitochondrial dehydrogenase reduction activity, and even an increase in its intensity. Moreover, in both cases the residual mitochondrial redox activity values were about twice the value found for the reference standard (50.3 ± 0.7%).

**Figure 7:**
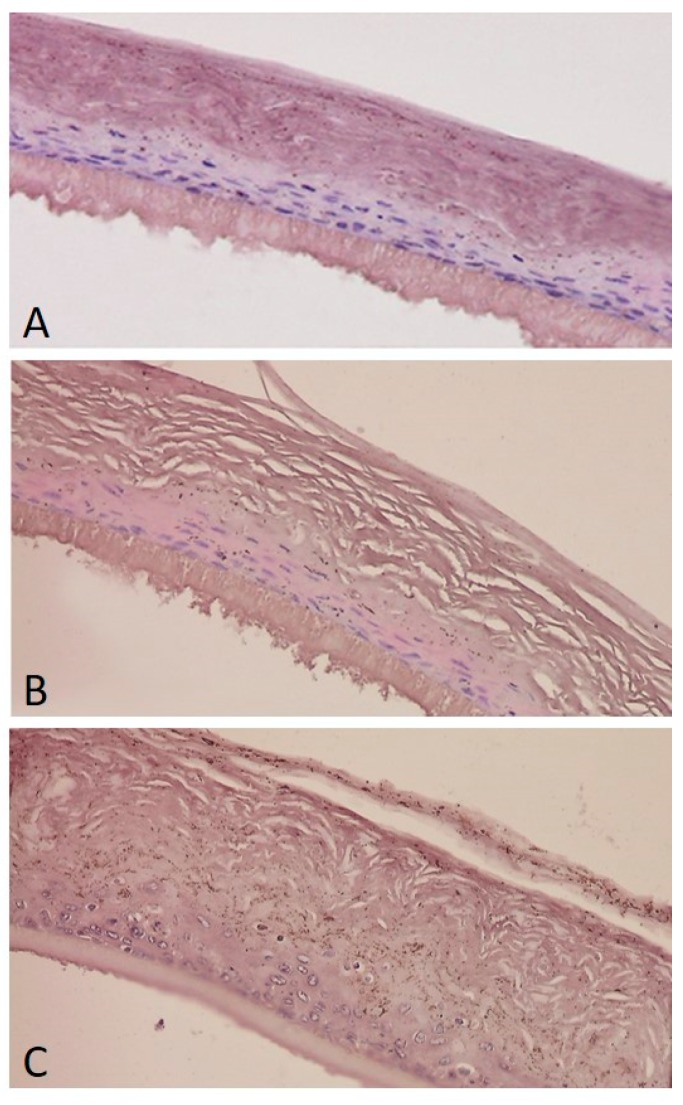
Histology of EpiSkin after exposure to ZLF-A (0.10%_w/w_)-enriched cream (**A**), ZLF-A extract (**B**), Triton ×100 (**C**); negative control). Hematoxilin-Eosin staining. ×400.

Histological examination was carried out on reconstructed human epidermis (RhE). Tissues exhibit from four to seven viable layers comprising at least basal, suprabasal, spinous and granular cell layers, and stratum corneum. According data from antiradical tests and MTT assay, histological observations of EpiSkin reconstructed tissue treated with ZLF-A (0.10%_w/w_)-enriched cream revealed a good tissue integrity with compacted stratum corneum (Figure 7A). Analogously, tissues’ preservation was observed after their exposure with ZLF-A extract alone (0.10%_w/w_). In this context, the stratum corneum retained its open basket-weave appearance (Figure 7B). Triton X-100 treatment defined tissue’s integrity loss and death (Figure 7C).

### 2.6. Preliminary in Vitro Stability Tests of ZLF-A Enriched Emulsion

During the formulation of a cosmetic product an investigation on its stability is necessary in order to guarantee the quality and safety for consumers in the foreseeable conditions of storage and use. Most cosmetic formulations are tested with emphasis on microbiological stability, whereas their physicochemical stability is only marginally investigated. Instead, a number of factors during the shelf-life of the product, such as temperature and pH changes, can affect the quality of the formulation. For a preliminary evaluation of the in vitro stability aliquots of 50 g of ZLF-A-enriched emulsion and of base cream were packaged in opaque white polyethylene flasks and stored at three different temperatures (4 ± 0.5 °C, 25 ± 2 °C and 37 ± 3 °C) and four storage periods (48 h, 7, 30 and 90 days). The room temperature (25 °C) was used to provide information about the behaviour of the product when stored under appropriate storage conditions, whereas the upper temperature mimics an accelerated aging. At each pre-determined time, samples underwent centrifugation, which produces stress in the sample, simulating an increase in gravity force and increasing the mobility of the particles, thus anticipating possible instabilities. These changes may appear in the form of precipitation, separation of phases, caking, or coalescence among others. Likely due to the proper homogenization during emulsion formulation [36], none of the cosmetic formulations exhibited phase separation, which evidenced their extremely high stability at mechanical stress, regardless of the storage temperature to which it has been exposed.

Monitoring the pH value is crucial for determining the emulsions’ stability. In fact, pH changes indicate the occurrence of chemical reactions that can give an idea on the quality of the final product [37]. The pH values of all the samples ranged from 4.15 ± 0.09 to 6.03 ± 0.08 and remained quite constant during storage, although there was a slight pH decrease after 7 days. It is generally recommended that skin care products should exhibit a pH of 5–5.5 to not disturb the physiological pH of the skin. In contrast to the viable epidermal cell layers, the stratum corneum is known to have an acidic pH between 4.1 and 5.8 under physiological conditions—the acid mantle—critical for the maintenance of the barrier homeostasis, regulation of cohesion/desquamation and antimicrobial defence. However, in certain conditions, e.g., dry or aged skin, there is a significant increase of skin pH leading to impaired barrier function and reduced buffer capacity, hence fostering the development of pathological skin conditions. The increased pH may be reversed by exogenous acidification of the stratum corneum [38]. In the light of the above considerations, the prepared emulsion is not only suitable for topical application, but even may be the best option to improve the acid mantle and skin barrier function. Indeed, Cucurbita pepo fruits are a common, high-value fruit vegetable [39], whose anti-genotoxicity and chemopreventive potential were recently explored [40]. The usage of pumpkin in cosmetic sector is not uncommon as its seeds, extracted by cold pressure, produce an oil rich in antioxidant and antimicrobial components. Tocopherols, sterols and polyunsaturated fatty acids in pumpkin oil were found to make it a cosmetic formula able to provide protection against dermatological wound [41].

## 3. Materials and Methods

### 3.1. Materials

All the solvents used for extraction and fractionation purposes, acetonitrile (LC-MS grade), formic acid (98%, for mass spectrometry), rutin, rebaudioside A, reagents for Folin–Ciocalteau, DPPH (2,2-diphenyl-1-picrylhydrazyl) and ABTS ([2,2-azinobis(3-ethylbenzothiazolin-6-sulfonic acid)] radical scavenging assays were purchased from Sigma-Aldrich (Buchs, Switzerland).

Cell culture media and reagents for cytotoxicity testing were purchased from Invitrogen (Paisley, Scotland, UK). MTT [3-(4,5-dimethyl-2-thiazolyl)-2,5-diphenyl-2*H*-tetrazolium bromide] was from Sigma-Aldrich Chemie GmbH. Episkin™ reconstructed human epidermis was purchased from Episkin (Lyon, France).

### 3.2. Plant Extraction and Fractionation

**Figure 8:**
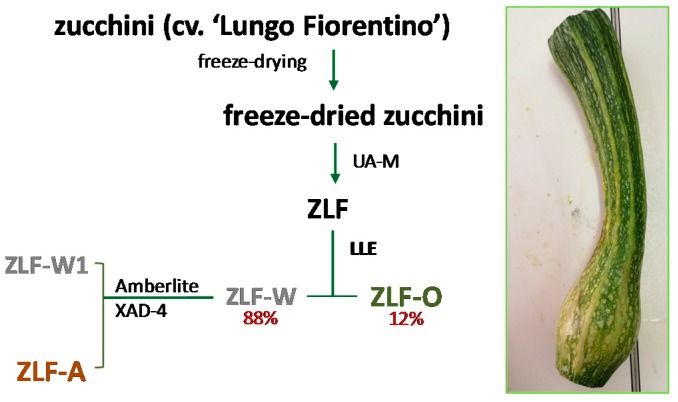
Extraction/fractionation scheme of *Cucurbita pepo* cv. ‘Lungo Fiorentino’. UA-M = Ultrasound Assisted Maceration; LLE = Liquid-Liquid Extraction.

Zucchini cv. ‘Lungo Fiorentino’ were harvested in July 2016 from a biological cultivation in Tuscany (latitude 43°42′32.0″ N and longitude 10°57′45.8″ E) and represent agro-wastes as they were out of gauge, and damaged by bad weather. The whole zucchini, once cleaned, underwent a freeze-drying procedure (FTS-System Flex-Dry, SP Scientific, Stone Ridge, NY, USA). 585 g of the lyophilized product were powdered by a rotary knife homogenizer and then wetted and subjected to maceration. To this end, three extraction cycles (30 min each) were performed in a 40 kHz ultrasonic bath (Branson M3800, Carouge, Switzerland) with 450 mL of EtOH for each sample. The parental extract obtained (ZLF, 10 g) was solubilised in water, and subsequently underwent discontinuous liquid–liquid extraction, using ethyl acetate as the extraction solvent. As a result, an organic fraction (ZLF-O) and an aqueous fraction (ZLF-W) were obtained. This latter was further chromatographed on Amberlite XAD-4 (h 70 cm, Ø 4.0 cm), eluting with water first (ZLF-W1) and then with MeOH (ZLF-A). The extraction/fractionation scheme is depicted in Figure 8.

### 3.3. UHPLC-HRMS Analyses

ZLF-O and ZLF-A fractions were investigated for their metabolic content by UHPLC-ESI-QqTOF-MS/MS techniques. To this purpose, a Shimadzu NEXERA UHPLC system was used with a Luna^®^ Omega Polar C18 column (1.6 μm particle size, 150 × 2.1 mm i.d., Phenomenex, Torrance, CA, USA). ZLF-O elution was achieved with a linear gradient of water (A) and acetonitrile (B), both with 0.1% formic acid under a linear gradient 5 → 95% B in 10 min, followed by 2 min of re-equilibration time in the initial conditions. A different linear gradient was used for the analysis of ZLF-A fraction: 0–5 min, 5 → 10% B; 5-10 min, 10 → 15% B; 10–12 min, 15 → 20% B; 12–13 min, 20 → 40% B; 13–15 min, 40% B. Then, also in this case the starting conditions were restored and the column was allowed to re-equilibrate for 2 min. In both cases a flow rate of 0.5 mL min^−1^ and an injection volume of 2.0 μL were set.

MS analysis was performed using the AB SCIEX TripleTOF 4600 (AB Sciex, Concord, ON, Canada) system with a DuoSpray™ ion source operating in negative electrospray ionisation. The APCI probe of the source was used for fully automatic mass calibration using the Calibrant Delivery System (CDS). CDS injects a calibration solution matching polarity of ionization and calibrates the mass axis of the TripleTOF^®^ system in all scan functions used (MS or MS/MS). Data were collected by information dependent acquisition (IDA) using parameters as in Table 3. The following parameter settings were also used: declustering potential (DP), 70 V; ion spray voltage, −4500 V; ion source heater, 600 °C; curtain gas, 35 psi; ion source gas, 60 psi. Data processing was performed using the PeakView^®^-Analyst^®^ TF 1.7 Software. Quantitative analysis of ZLF-A identified constituents was also carried out. Rutin (y = 125837x + 26331; R^2^ = 0.9998) was used as reference standard for identified glycosylated flavonoids and rebaudioside A (y = 208034x − 1638.2; R^2^ = 1) for the ent-kaurene diterpene glycoside. Calibration curves of both the standards were constructed, based on analyses performed in the same experimental conditions of ZFL-A fraction. Results were expressed as mean ± SD values of three independent measurements.

### 3.4. Determination of Total Phenols

Total phenol amount of ZLF parental extract and of the fractions derived was determined according to the Folin–Ciocalteau procedure [42] with slight modifications. Analyzed samples (0.25 and 0.50 mg/mL in DMSO) were mixed with 0.250 mL of the Folin–Ciocalteau reagent (FCR) and 2.25 mL of Na_2_CO_3_ (7.5% *w*/*v*). After stirring the reaction mixture at room temperature for 2 h, 300 µL of each sample were transferred into a multiwell plate and the absorbance was read at 765 nm using a Wallac Victor^3^ multilabel plate reader (PerkinElmer Inc., Waltham, MA, USA). The content of total phenols of the samples was expressed as milligram gallic acid equivalents (GAEs) per g of dried extract ± standard deviation (SD).

### 3.5. Determination of DPPH Radical Scavenging Capacity

DPPH radical scavenging capability was determined as previously reported [43] with slight modifications. Briefly, ZLF-O and ZLF-A, tested at different final concentration levels (12.5, 25.0, 50.0 and 100.0 μg/mL), were dissolved in a DPPH^•^ methanol solution (9.4 × 10^−5^ M) at room temperature. After stirring the reaction mixture at room temperature for 20 min, 300 µL of each sample were transferred into a multiwell plate and the absorbance was read at 520 nm using a Wallac Victor^3^ multilabel plate reader (PerkinElmer Inc., Waltham, MA, USA). Results are the mean ± SD values. ID_50_, based on the percentage decrease of the initial DPPH^•^ absorption by the different concentrations of the test samples, and TEAC (Trolox^®^ Equivalent Antioxidant Capacity) values were also calculated.

### 3.6. Determination of ABTS Radical Cation Scavenging Capacity

ABTS radical cation was generated by reacting ABTS (7.0 mM) and K_2_S_2_O_8_ (2.45 mM). The mixture was allowed to stand in the dark at room temperature for 16 h, and then diluted with PBS (pH 7.4) in order to reach an absorbance of 0.70 at 734 nm. ZLF-O and ZLF-A extracts (12.5, 25.0, 50.0 and 100.0 μg/mL, final concentration levels) were dissolved in 1.0 mL of diluted ABTS^●+^ solution. After 6 min of incubation, 300 µL of each sample were transferred into a multiwell plate and the absorbance was read at 734 nm using a Wallac Victor^3^ multilabel plate reader (PerkinElmer Inc., Waltham, MA, USA). Results, expressed in terms of the percentage decrease of the initial ABTS^●+^ absorption by the test samples, are the mean ± SD values [44]. ID_50_ and TEAC values were also calculated.

### 3.7. Cell culture and Cytotoxicity Assessment

Human HaCat keratinocyte were grown in RPMI 1600 high glucose medium supplemented with 10% Newborn Calf Serum, 50.0 U/mL penicillin, and 100.0 μg/mL streptomycin, at 37 °C in a humidified atmosphere containing 5% CO_2_. Human MRC-5 lung fibroblasts cell lines were grown in the same conditions except for the medium, which was DMEM supplemented with 10% Fetal Bovine Serum. Cells were seeded in 96-multiwell plates at a density of 1.5 × 10^4^ cells/well. After 24 h cells were treated with ZLF-O and ZLF-A extracts at 25.0, 50.0, 100.0, 200.0 and 500.0 μg/mL final dose levels. At 24, 48 and 72 h of incubation, inhibition of mitochondrial redox activity was determined by MTT cell viability test as previously described [45].

### 3.8. Radical Scavenging Capacity of Zucchini-Based Cosmeceutical Emulsions

#### 3.8.1. Cream Formulation

Base cream was prepared as an oil-in-water disperse system by heating separately the aqueous phase (45%) and the oily phase containing white wax (5%) and sweet almond oil (40%). ZLF-O and ZLF-A extracts, previously solubilized in pure ethanol, were incorporated into the base cream in two different amounts (0.010 and 0.10%_w/w_). Then, the emulsion was further enriched with ascorbic acid (0.08%), quercetin (0.005%) and *Lavandula officinalis* L. essential oil (3.0%) for antioxidant, preservative and antimicrobic purposes.

#### 3.8.2. Determination of Antiradical Activity

Base cream and the emulsions containing zucchini extracts were tested for their scavenging efficacy towards DPPH^•^ and ABTS^•+^. Samples were prepared with a protocol similar to that previously described by Pelle et al. [46]. Briefly, emulsions were dissolved in iPrOH (1% *w*/*v*) and 5-fold diluted with distilled water. Then, the radical probe (DPPH^•^ or ABTS^●+^) was added and the spectrophotometric analysis were carried out as described for pure extracts (see Section 2.5 and Section 2.6).

#### 3.8.3. Cytotoxicity Assessment in an In Vitro Reconstructed Human Epidermis (EpiSkin™)

Reconstructed Human Epidermis (small, age day 17–0.50 cm^2^) was removed from the agarose nutritive solution provided by the manufacturer, transferred in a 6-well plate containing fresh growth medium and incubated at 37 °C in a humidified atmosphere containing 5% CO_2_. After 24 h a small amount (60 µg/cm^2^) of the base cream or of that enriched with ZLF-A extract was deposited onto the surface of the tissues and spread with a small paintbrush. The cultures were incubated at 37 °C for 24 h, after which mitochondrial redox activity inhibition was determined by MTT test.

#### 3.8.4. Histological Analysis on Reconstructed Human Epidermis (EpiSkin™)

An important EpiSkin quality control criteria is based on histological examination [47]. After the treatment, tissues were rapidly immersed in Bouin’s fluid. The samples were dehydrated through a graded ethanol series and finally embedded in paraffin. Then, paraffin sections (5 µm thickness) were stained with haematoxylin and eosin stain and examined under a light microscope (Leica DM LB) for histological evaluation. Photographs were taken using the Leica ICC50 HD digital camera [48,49,50].

### 3.9. Stability Tests

Stability tests were performed at different conditions, predictive of different climatic conditions, in order to explore their effects on the emulsions’ storage. To this aim, samples were kept at 4 ± 0.5 °C, 25 ± 2 °C and 37 ± 3 °C. Phase separation was evaluated after 48 h, 7, 30 and 90 days of storage, centrifuging 1 g of each sample at 1500 rpm for 10 min, then at 300 rpm for 10 min and at 4000 rpm for 5 min using an Allegra™ 64R centrifuge (Beckman, Palo Alto, CA, USA), equipped with a F2402H rotor. The pH measurements were repeated at each storage time on all the emulsions kept at different conditions (1 g dissolved in 15 mL of pure water).

## 4. Conclusions

Fruits and vegetables, with their undeniable nutritional and nutraceutical value, are an essential part of daily diet, but agricultural processing, in spite of what comes on our tables, mainly based on consumer’s demand, generates abundant raw materials, which can range from whole, and unexploited materials to fractions and/or mixtures produced by physical, thermal, chemical processing. Indeed, vegetable and fruit trimmings could be more valuable than the main products [51], thanks to their diversity in sugars, minerals, organic acids, dietary fibres and bioactive compounds, which could be similar to that in their edible and pleasantly palatable counterparts. Herein, the exploitation and re-evaluation of zucchini cv. ‘Lungo Fiorentino’ wastes, through ultrasound assisted maceration, an eco-friendly extraction processes, represent a valuable alternative to recycle disposable products in green cosmetics. The application of UHPLC-MRMS analytical method to identify polyphenols and other polar, as well as apolar compounds, for the first time, in *Cucurbita pepo* cv. ‘Lungo Fiorentino’ allowed the deep fruit investigation for its nutraceutical and cosmeceutical purposes. The recovery of fine and precious chemicals from zucchini wastes was in accordance with valorisation and sustainable development principles, and was prompt to convey a new skin care concept through products in which the nutraceutical zucchini value was further exploited for its cosmeceutical strength.

## Figures and Tables

**Table 1:** TOF and TOF-MS^2^ data of compounds tentatively identified in ZLF-A fraction.

Peak n.	Rt (min)	Tentative Assignment	Formula	[M−H]^−^ Found (*m*/*z*)	[M−H]^−^ Calc. (*m*/*z*)	Error (ppm)	RDB	MS/MS Fragment Ions (*m*/*z*)
**1**	8.80	Myricetin 3-*O*-hexoside	C_21_H_20_O_13_	479.0826	479.0831	−1.0	12	317.0313; 316.0218; 287.0194; 271.0234; 259.0247
**2**	9.33	Quercetin 3-*O*-dideoxyhexosyl-hexoside	C_33_H_40_O_20_	755.2025	755.2040	−2.0	14	609.1484; 591.1386; 489.1059; 301.0355; 300.0277; 271.0246; 255.0297; 178.9985
**3**	9.71	Quercetin 3-*O*-hexosyl-pentoside	C_26_H_28_O_16_	595.1303	595.1304	−0.2	13	301.0350; 300.0279; 287.0578; 271.0249; 255.0292
**4**	10.64	Kaempferol 3-*O*-(2′′,6′′-di-*O*-deoxyhexosyl)hexoside (e.g., clitorin)	C_33_H_40_O_19_	739.2066	739.2091	−3.4	14	575.1429; 285.0406; 284.0325; 255.0300; 227.0350
**5**	10.72	Rutin	C_27_H_30_O_16_	609.1462	609.1461	−0.2	13	343.0461; 301.0351; 300.0271; 271.0245; 255.0298; 243.0297; 178.9980; 151.0031
**6**	10.97	Quercetin 3-*O*-hexoside	C_21_H_20_O_12_	463.0876	463.0882	1.3	12	301.0360; 300.0280; 271.0252; 255.0302; 243.0297; 151.0032
**7**	10.98	Isorhamnetin 3-*O*-(2″,6″-di-*O*-deoxyhexosyl) hexoside (e.g., typhaneoside)	C_34_H_42_O_20_	769.2183	769.2197	−1.8	14	605.1556; 315.0514; 314.0439; 299.0204; 271.0252; 243.0301
**8**	11.19	Luteolin hexoside	C_21_H_20_O_11_	447.0925	447.0933	−1.8	12	285.0395; 284.0314
**9**	11.20	Luteolin hexosyl-deoxyhexoside	C_27_H_30_O_15_	593.1506	593.1512	−1.0	13	285.0404; 284.0314
**10**	11.36	Kaempferol hexosyl-deoxyhexoside (isomer 1)	C_27_H_30_O_15_	593.1514	593.1512	0.3	13	473.1109; 447.0953; 429.0826; 327.0503; 285.0395; 284.0322; 255.0292; 227.0341; 178.9978; 151.0037
**11**	11.41	Kaempferol 3-*O*-hexosyl-pentoside (e.g., sambubioside)	C_26_H_28_O_15_	579.1358	579.1355	0.4	13	357.1471; 285.0387; 284.0315; 255.0287; 227.0331
**12**	11.65	Isorhamnetin 4′-*O*-rutinoside	C_28_H_32_O_16_	623.1619	623.1618	0.2	13	477.1032; 459.0941; 443.2295; 357.0613; 339.0506; 315.0505; 314.0428; 299.0188; 285.0386; 271.0238; 255.0287; 243.0292; 227.0332; 199.0390; 178.9975; 151.0021
**13**	12.27	Kaempferol hexosyl-deoxyhexoside (isomer 2)	C_27_H_30_O_15_	593.1520	593.1512	1.4	13	327.0513; 285.0404; 284.0327; 257.0455; 255.0299; 227.0351
**14**	12.27	Kaempferol 3-*O*-hexoside	C_21_H_20_O_11_	447.0924	447.0933	−2.4	12	285.0392; 284.0326; 257.0437; 255.0302; 227.0342
**15**	12.60	Isorhamnetin 7-*O*-rutinoside	C_28_H_32_O_16_	623.1620	623.1618	0.4	13	357.0623; 315.0514; 314.0438; 300.0282; 299.0188; 285.0409; 271.0253; 255.0300; 243.0304
**16**	13.31	Anthraquinone derivative 1	C_34_H_36_O_17_	715.1893	715.1880	1.8	17	621.1469; 407.0773; 406.0694; 313.0346; 312.0269; 285.0381; 283.0243
**17**	13.53	Anthraquinone derivative 2	C_34_H_36_O_16_	699.1942	699.1931	1.6	17	605.1517; 391.0806; 390.0731; 333.0745; 297.0385; 296.0304; 269.0432; 267.0276
**18**	13.73	Ent-kaurene diterpene glycoside	C_38_H_60_O_18_	803.3713	803.3707	0.8	9	641.3231 (→479.2690; 461.2578; 335.2240; 317.2119); 623.3117; 479.2673; 413.2344; 317.2129

**Rt** = retention time; **[M**−**H]^−^** found (*m*/*z*) = deprotonated ions experimentally observed in TOF-MS spectra (*m*/*z* of the monoisotopic peaks); **[M**−**H]**^−^ calc. (*m*/*z*) = theoretical deprotonated ions for a given molecular formula (*m*/*z* of the monoisotopic peaks); **error (ppm)** = mass error of an assignment, obtained comparing a theoretical *m*/*z* and an experimentally observed *m*/*z* (accepted values < 5 ppm); **RDB** = Rings and Double Bonds (conventional measure of the degree of unsaturation).

**Table 2:** TOF-MS and TOF-MS^2^ data of compounds putatively identified in ZLF-O fraction.

Peak n.	Rt (min)	Tentative Assignment	Formula	[M−H]^−^ Found (*m*/*z*)	[M−H]^−^ Calc. (*m*/*z*)	Error (ppm)	RDB	MS/MS Fragment Ions (*m*/*z*)
**1**	2.420	*p*-Coumaric acid	C_9_H_8_O_3_	163.0407	163.0401	3.9	6	119.0508; 117.0348; 93.0351
**2**	2.557	Quercetin rutinoside	C_27_H_30_O_16_	609.1464	609.1461	0.5	13	301.0349; 300.0275; 271.0247; 255.0297
**3**	2.674	Quercetin hexoside	C_21_H_20_O_12_	463.0871	463.0882	−2.4	12	301.0347; 300.0279; 271.0241; 255.0298; 151.0026
**4**	2.791	Kaempferol rutinoside	C_27_H_30_O_15_	593.1520	593.1512	1.4	13	285.0407; 284.0327; 255.0296
**5**	2.85	(iso)rhamnetin rutinoside	C_28_H_32_O_16_	623.1620	623.1618	0.4	13	315.0519; 314.0435; 300.0279; 299.0204; 271.0248
**6**	2.928	Quercetin deoxyhexoside	C_21_H_20_O_11_	447.0929	447.0933	−0.9	12	301.0350; 300.0274; 271.0245; 255.0298; 243.0300
**7**	3.240	Kaempferol deoxyhexoside	C_21_H_20_O_10_	431.0984	431.0984	0.1	12	285.0413; 284.0326; 255.0303; 227.0350
**8**	3.530	Cartamidin	C_15_H_12_O_6_	287.0560	287.0561	−0.4	10	151.0027; 135.0449; 134.0371
**9**	3.630	Quercetin	C_15_H_10_O_7_	301.0351	301.0354	−0.9	11	273.0406; 245.0448; 227.0351; 178.9982; 151.0037; 121.0295; 107.0141
**10**	3.668	Oxo-dihydroxyoctadecenoic acid hexoside	C_24_H_42_O_10_	489.2708	489.2705	0.6	4	327.2179; 291.1973; 229.1445; 211.1338; 171.1026
**11**	4.174	Trihydroxyoctadecadienoic acid 1	C_18_H_32_O_5_	327.2177	327.2177	0.0	3	309.2078; 291.1966; 229.1448; 211.1347; 183.1396; 171.1034; 165.1290; 137.0977
**12**	4.405	Trihydroxyoctadecenoic acid	C_18_H_34_O_5_	329.2335	329.2333	0.5	2	311.2232; 293.2124; 229.1449; 211.1347; 183.1396; 171.1029; 127.1130
**13**	4.521	n.i.	C_18_H_32_O_5_	327.2174	327.2177	−0.9	3	291.1970; 239.1655; 221.1546; 197.1181; 195.1390; 179.1437
**14**	4.690	n.i.	C_13_H_18_O_4_	237.1137	237.1132	2.0	5	217.0877; 193.1242; 177.0927; 165.0925; 133.1024
**15**	5.222	n.i.	C_25_H_38_O_6_	433.2600	433.2596	1.0	7	327.2184; 291.1967; 229.1441; 211.1337; 201.1123; 183.1388; 171.1023
**16**	5.320	Trihydroxyoctadecadienoic acid 2	C_18_H_32_O_5_	327.2177	327.2177	−0.3	3	309.2078; 291.1967; 283.1931; 265.1820; 239.1643; 211.1336; 183.1387; 171.1025; 135.0455
**17**	5.533	Dihydroxyoctadecadienoic acid	C_18_H_32_O_4_	311.2232	311.2228	1.3	3	293.2128; 275.2018; 235.1702; 223.1706; 201.1134; 199.0974; 171.1025; 165.0919; 155.1078; 127.1129; 125.0974
**18**	5.726	n.i.	C_24_H_34_O_6_	417.2288	417.2283	1.3	8	373.2402; 301.2179; 259.1711
**19**	5.803	n.i.	C_17_H_26_O_4_	293.1764	293.1758	1.9	5	249.1863; 193.1599; 192.1160; 177.0922; 136.0897; 121.0657
**20**	5.899	Dihydroxyoctadecenoic acid 1	C_18_H_34_O_4_	313.2388	313.2384	1.2	2	295.2287; 277.2175; 201.1135; 195.1393; 183.1393; 129.0921; 99.0818
**21**	5.956	Dihydroxyoctadecenoic acid 2	C_18_H_34_O_4_	313.2392	313.2384	2.4	2	295.2285; 277.2177; 201.1135; 199.0975; 171.1029; 165.0922; 155.1082; 127.1133; 125.0974
**22**	6.494	Hydroxyoctadecatrienoic acid	C_18_H_30_O_3_	293.2126	293.2122	1.3	4	275.2022; 223.1334; 205.1219; 195.1387; 183.1383; 171.1023; 121.1020
**23**	7.985	Hydroxypalmitic acid	C_16_H_32_O_3_	271.2283	271.2279	1.6	1	253.2175; 225.2228; 223.2070; 221.1912; 197.1909
**24**	8.187	Linolenic acid	C_18_H_30_O_2_	277.2179	277.2173	2.1	4	259.2089; 127.0774
**25**	8.665	Linoleic acid	C_18_H_32_O_2_	279.2334	279.2330	1.6	3	261.2227
**26**	9.023	Palmitic acid	C_16_H_32_O_2_	255.2337	255.2330	2.9	1	237.2214; 201.8350; 166.8665

**Rt***=* retention time; **[M−H]^−^** found (***m*/*z***) *=* deprotonated ions experimentally observed in TOF-MS spectra (*m*/*z* of the monoisotopic peaks)*;* **[M−H]^−^ calc.** (***m*/*z***) *=* theoretical deprotonated ions for a given molecular formula (*m*/*z* of the monoisotopic peaks); **error (ppm)** = mass error of an assignment, obtained comparing a theoretical *m*/*z* and an experimentally observed *m*/*z* (accepted values < 5 ppm); **RDB** = Rings and Double Bonds (conventional measure of the degree of unsaturation); **n.i.** = not identified.

**Table 3:** Information dependent acquisition (IDA) parameters applied in HR MS/MS analysis.

	ZLF-O	ZLF-A
**TOF-MS Survey Scan Range**	100–1500 Da	250–950 Da
**TOF-MS Accumulation Time**	250 ms	250 ms
**TOF-MS/MS Scan Range**	80–1250 Da	100–800 Da
**TOF-MS/MS Accumulation Time**	100 ms	100 ms
**Collision Energy**	45 V	35 V
**Collision Energy Spread**	15 V	25 V
**Declustering Potential**	60 V	70 V

## Supplementary Materials

The following are available online, Figure S1: TOF-MS^2^ spectrum of compound **2** and the hypothesized structure of the product ion at *m*/*z* 609.1484, Figure S2: (a) TOF-MS^2^ spectra of metabolites **16** and **17**; (b) Proposed fragmentation pathway (measured exact mass of each fragment ion, as listed in Table 2, was at *m*/*z* value to within 5 ppm vs. its relative theoretical *m*/*z* value. This latter was reported below each structure), Figure S3: TOF-MS spectra of (a) the ent-kaurene diterpene glycoside (compound **18**) tentatively identified in ZLF-A fraction; (c) rebaudioside A commercial standard. TOF-MS^2^ spectra of (b) the ent-kaurene diterpene glycoside (compound **18**) tentatively identified in ZLF-A fraction; (d) rebaudioside A commercial standard, Table S1: Quantitative data of ZLF-A identified constituents.

## References

[1] Barbulova A., Colucci G., Apone F. (2015). New Trends in Cosmetics: By-Products of Plant Origin and Their Potential Use as Cosmetic Active Ingredients. Cosmetics.

[2] Ribeiro A.S., Estanqueiro M., Oliveira M.B., Sousa Lobo J.M. (2015). Main Benefits and Applicability of Plant Extracts in Skin Care Products. Cosmetics.

[3] Piccolella S., Pacifico S., Fishbein J.C., Heilman J.M. (2015). Plant-Derived Polyphenols: A Chemopreventive and Chemoprotectant Worth-Exploring Resource in Toxicology. Advances in Molecular Toxicology.

[4] Nichols J.A., Katiyar S.K. (2009). Skin photoprotection by natural polyphenols: Anti-inflammatory, antioxidant and DNA repair mechanisms. Arch. Dermatol. Res..

[5] Zillich O.V., Schweiggert-Weisz U., Eisner P., Kerscher M. (2015). Polyphenols as active ingredients for cosmetic products. Int. J. Cosmet. Sci..

[6] Lin T.K., Zhong L., Santiago J.L. (2017). Anti-Inflammatory and Skin Barrier Repair Effects of Topical Application of Some Plant Oils. Int. J. Mol. Sci..

[7] Ghosh P.R., Fawcett D., Sharma S.B., Poinern G.E. (2016). Progress towards Sustainable Utilisation and Management of Food Wastes in the Global Economy. Int. J. Food Sci..

[8] Zuin V.G., Ramin L.Z. (2018). Green and Sustainable Separation of Natural Products from Agro-Industrial Waste: Challenges, Potentialities, and Perspectives on Emerging Approaches. Top. Curr. Chem. (Cham).

[9] Omar S.H. (2010). Oleuropein in olive and its pharmacological effects. Sci. Pharm..

[10] Rodrigues F., Pimentel F.B., Oliveira M.B.P.P. (2015). Olive by-products: Challenge application in cosmetic industry. Ind. Crop Prod..

[11] Galanakis C.M., Tsatalas P., Galanakis I.M. (2018). Implementation of phenols recovered from olive mill wastewater as UV booster in cosmetics. Ind. Crop. Prod..

[12] Kang G.J., Han S.C., Yi E.J., Kang H.K., Yoo E.S. (2011). The Inhibitory Effect of Premature *Citrus unshiu* Extract on Atopic Dermatitis In Vitro and *In Vivo*. Toxicol Res..

[13] Kang G.J., Han S.C., Ock J.W., Kang H.K., Yoo E.S. (2013). Anti-Inflammatory Effect of Quercetagetin, an Active Component of Immature *Citrus unshiu*, in HaCaT Human Keratinocytes. Biomol. Ther. (Seoul).

[14] Teixeira A., Baenas N., Dominguez-Perles R., Barros A., Rosa E., Moreno D.A., Garcia-Viguera C. (2014). Natural bioactive compounds from winery by-products as health promoters: A review. Int. J. Mol. Sci..

[15] Murthy S., Naidu M. (2012). Sustainable management of coffee industry by-products and value addition—A review. Resour. Conserv. Recycl..

[16] Johnson L.K., Dunning R., Bloom J.D.D., Gunter C.C., Boyette M.D., Creamer N.G. (2018). Estimating on-farm food loss at the field level: A methodology and applied case study on a North Carolina farm. Resour. Conserv. Recycl..

[17] Global Food Losses and Food Waste—Extent, Causes and Prevention. http://www.fao.org/docrep/014/mb060e/mb060e00.pdf.

[18] Pacifico S., Piccolella S., Nocera P., Tranquillo E., Dal Poggetto F., Catauro M. (2019). New insights into phenol and polyphenol composition of *Stevia rebaudiana* leaves. J. Pharm. Biomed. Anal..

[19] Iswaldi I., Gómez-Caravaca A.M., Lozano-Sánchez J., Arráez-Román D., Segura-Carretero A., Fernández-Gutiérrez A. (2013). Profiling of phenolic and other polar compounds in zucchini (*Cucurbita pepo* L.) by reverse-phase high-performance liquid chromatography coupled to quadrupole time-of-flight mass spectrometry. Food Res. Int..

[20] Tsiklauri L., An G., Ruszaj D.M., Alaniya M., Kemertelidze E., Morris M.E. (2011). Simultaneous determination of the flavonoids robinin and kaempferol in human breast cancer cells by liquid chromatography-tandem mass spectrometry. J. Pharm. Biomed. Anal..

[21] Faugno S., Piccolella S., Sannino M., Principio L., Crescente G., Baldi G.M., Fiorentino N., Pacifico S. (2019). Can agronomic practices and cold-pressing extraction parameters affect phenols and polyphenols content in hempseed oils?. Ind. Crop Prod..

[22] Abu-Reidah I.M., Arráez-Román D., Quirantes-Piné R., Fernández-Arroyo S., Segura-Carretero A., Fernández-Gutiérrez A. (2012). HPLC–ESI-Q-TOF-MS for a comprehensive characterization of bioactive phenolic compounds in cucumber whole fruit extract. Food Res. Int..

[23] Pacifico S., Galasso S., Piccolella S., Kretschmer N., Pan S.-P., Nocera P., Lettieri A., Bauer R., Monaco P. (2018). Winter wild fennel leaves as a source of anti-inflammatory and antioxidant polyphenols. Arab. J. Chem..

[24] Brahmi-Chendouh N., Piccolella S., Crescente G., Pacifico F., Boulekbache L., Hamri-Zeghichi S., Akkal S., Madani K., Pacifico S. (2019). A nutraceutical extract from *Inula viscosa* leaves: UHPLC-HR-MS/MS based polyphenol profile, and antioxidant and cytotoxic activities. J. Food Drug Anal..

[25] Yasir M., Sultana B., Nigam P.S., Owusu-Apenten R. (2016). Antioxidant and genoprotective activity of selected cucurbitaceae seed extracts and LC–ESIMS/MS identification of phenolic components. Food Chem..

[26] Nikaido T., Ohmoto T., Sankawa U., Kitanaka S., Takido M. (1984). Inhibitors of adenosine 3′, 5′-cyclic monophosphate phosphodiesterase in Cassia seed. Chem. Pharm. Bull..

[27] Singh J., Singh J. (1987). Two anthraquinone glycosides from *Cassia marginata* roots. Phytochemistry.

[28] Rai K.N., Ranjan S., Chandra S.S. (2009). Isolation and characterization of anthraquinone derivatives from the heartwood of *Cassia glauca* Lam. Asian J. Chem..

[29] Pacifico S., Piccolella S., Nocera P., Tranquillo E., Dal Poggetto F., Catauro M. (2017). Steviol glycosides content in cultivated *Stevia rebaudiana* Bertoni: A new sweet expectation from the Campania region (Italy). J. Food Compos. Anal..

[30] García P.A., De Oliveira A.B., Batista R. (2007). Occurrence, biological activities and synthesis of kaurane diterpenes and their glycosides. Molecules.

[31] Kikuchi T., Ando H., Maekawa K.I., Arie H., Yamada T., Tanaka R. (2015). Two new ent-kaurane-type diterpene glycosides from zucchini (*Cucurbita pepo* L.) seeds. Fitoterapia.

[32] Bang M.H., Han J.T., Kim H.Y., Park Y.D., Park C.H., Lee K.R., Baek N.I. (2002). 13-Hydroxy-9Z,11E,15E-octadecatrienoic acid from the leaves of *Cucurbita moschata*. Arch. Pharm. Res..

[33] Cotovio J., Grandidier M.-H., Lelièvre D., Roguet R., Tinois-Tessonneaud E., Leclaire J. (2008). In vitro acute skin irritancy of chemicals using the validated EPISKIN model in a tiered strategy. Results and performances with 184 cosmetic ingredients. AATEX.

[34] EU (2008) Council Regulation (EC) No 440/2008 of 30 May 2008 Laying down Test Methods Pursuant to Regulation (EC) No 1907/2006 of the European Parliament and of the Council on the Registration, Evaluation, Authorisation and Restriction of Chemicals (REACH). https://eur-lex.europa.eu/legal-content/EN/ALL/?uri=celex:32008R0440.

[35] (2015). Test No. 439: In Vitro Skin Irritation: Reconstructed Human Epidermis Test Method.

[36] Nour A.H., Yunus R.M. (2006). Stability investigation of water-in-crude oil emulsion. J. Appl. Sci..

[37] Smaoui S., Hlima H.B., Chobba I.B., Kadri A. (2017). Development and stability studies of sunscreen cream formulations containing three photo-protective filters. Arabian J. Chem..

[38] Abels C., Angelova-Fischer I. (2018). Skin Care Products: Age-Appropriate Cosmetics. Curr. Probl. Dermatol..

[39] Lust T.A., Paris H.S. (2016). Italian horticultural and culinary records of summer squash (*Cucurbita pepo*, Cucurbitaceae) and emergence of the zucchini in 19th-century Milan. Ann. Bot..

[40] Martínez-Valdivieso D., Font R., Fernández-Bedmar Z., Merinas-Amo T., Gómez P., Alonso-Moraga Á., Del Río-Celestino M. (2017). Role of Zucchini and Its Distinctive Components in the Modulation of Degenerative Processes: Genotoxicity, Anti-Genotoxicity, Cytotoxicity and Apoptotic Effects. Nutrients.

[41] Bardaa S., Ben Halima N., Aloui F., Ben Mansour R., Jabeur H., Bouaziz M., Sahnoun Z. (2016). Oil from pumpkin (*Cucurbita pepo* L.) seeds: Evaluation of its functional properties on wound healing in rats. Lipids Health Dis..

[42] Pacifico S., Piccolella S., Galasso S., Fiorentino A., Kretschmer N., Pan S.-P., Bauer R., Monaco P. (2016). Influence of harvest season on chemical composition and bioactivity of wild rue plant hydroalcoholic extracts. Food Chem. Toxicol..

[43] Pacifico S., Piccolella S., Marciano S., Galasso S., Nocera P., Piscopo V., Fiorentino A., Monaco P. (2014). LC-MS/MS profiling of a mastic leaf phenol enriched extract and its effects on H_2_O_2_ and Aβ (25–35) oxidative injury in SK-B-NE (C)-2 cells. J. Agric. Food Chem..

[44] Di Maro A., Pacifico S., Fiorentino A., Galasso S., Gallicchio M., Guida V., Severino V., Monaco P., Parente A. (2013). Raviscanina wild asparagus (*Asparagus acutifolius* L.): A nutritionally valuable crop with antioxidant and antiproliferative properties. Food Res. Int..

[45] Piccolella S., Nocera P., Carillo P., Woodrow P., Greco V., Manti L., Fiorentino A., Pacifico S. (2016). An apolar *Pistacia lentiscus* L. leaf extract: GC-MS metabolic profiling and evaluation of cytotoxicity and apoptosis inducing effects on SH-SY5Y and SK-N-BE(2)C cell lines. Food Chem. Toxicol..

[46] Pelle E., Mammone T., Marenus K., Dicanio D., Maes D. (2002). A test for antioxidant activity in cosmetic formulations. J. Cosmet. Sci..

[47] Pellevoisin C., Videau C., Briotet D., Grégoire C., Tornier C., Alonso A., Rigaudeau A.S., Bouez C., Seyler N. (2018). SkinEthic™ RHE for in vitro evaluation of skin irritation of medical device extracts. Toxicol. In Vitro.

[48] Rosati L., Prisco M., Di Fiore M.M., Santillo A., Sciarrillo R., Valiante S., Laforgia V., Coraggio F., Andreuccetti P., Agnese M. (2015). Sex steroid hormone secretion in the wall lizard *Podarcis sicula* testis: The involvement of VIP. J. Exp. Zool. A Ecol. Genet. Physiol..

[49] Di Fiore M.M., Burrone L., Santillo A., Chieffi Baccari G., Tohru Yoshimura T., Nishikawa T., Homma H. (2016). Endocrine Activity of D-Aspartate in Nonmammalian Animals. D-Amino Acids. Physiology, Metabolism, and Application.

[50] Santillo A., Falvo S., Chieffi G., Di Fiore M.M. (2017). Seasonal changes in gene expression of steroidogenic enzymes, androgen and estrogen receptors in frog testis. Acta Zool..

[51] Varzakas T., Zakynthinos G., Verpoort F. (2016). Plant Food Residues as a Source of Nutraceuticals and Functional Foods. Foods.

