# Supplementary material for: Recovering Cucurbita pepo cv. ‘Lungo Fiorentino’ Wastes: UHPLC-HRMS/MS Metabolic Profile, the Basis for Establishing Their Nutra- and Cosmeceutical Valorisation
Source: Molecules. 2019 Apr 15;24(8):1479. doi: 10.3390/molecules24081479 (PMC6514934; doi:10.3390/molecules24081479)
Supplement: Supplementary file 1 [file molecules-24-01479-s001.pdf]

Recovering *Cucurbita pepo* cv. 'Lungo Fiorentino' wastes: UHPLC-HRMS/MS metabolic profile, the basis for establishing their nutra- and cosmeceutical valorisation

Simona Piccolella,[†] Alessandro Bianco,[†] Giuseppina Crescente, Alessandra Santillo, Gabriella Chieffi Baccari and Severina Pacifico [*]

Department of Environmental Biological and Pharmaceutical Sciences and Technologies, University of Campania "Luigi Vanvitelli", Via Vivaldi 43, I-81100 Caserta, Italy;

* Correspondence: severina.pacifico@unicampania.it; Tel.: +39-0823-274578

Supplementary Information

Figure S1. TOF-MS² spectrum of compound **2** and the hypothesized structure of the product ion at *m*/*z* 609.1484.

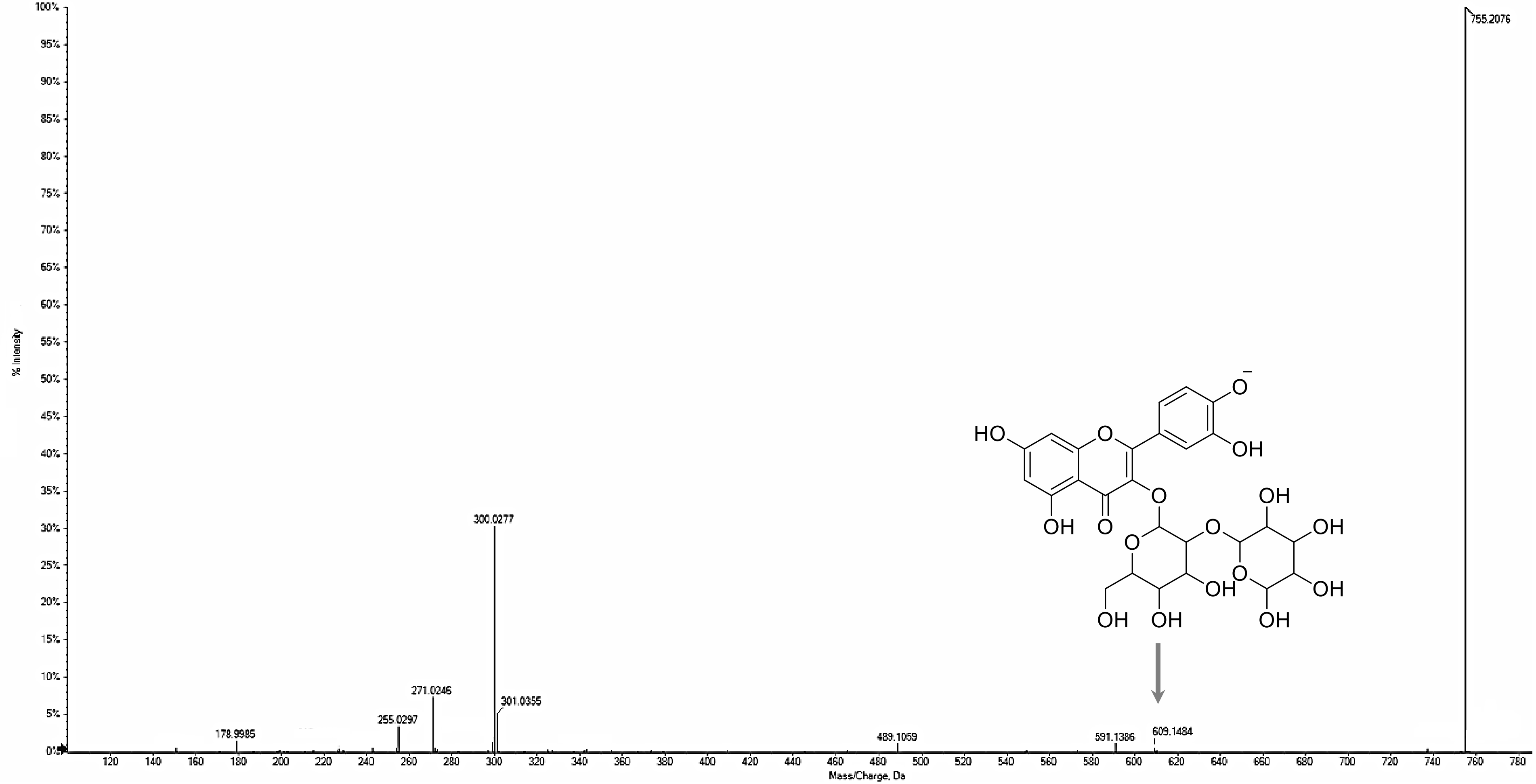

Figure S2. (a) TOF-MS² spectra of metabolites **16** and **17**; (b) Proposed fragmentation pathway (measured exact mass of each fragment ion, as listed in Table 2, was at *m/z* value to within 5 ppm *vs*. its relative theoretical *m/z* value. This latter was reported below each structure).

(a)

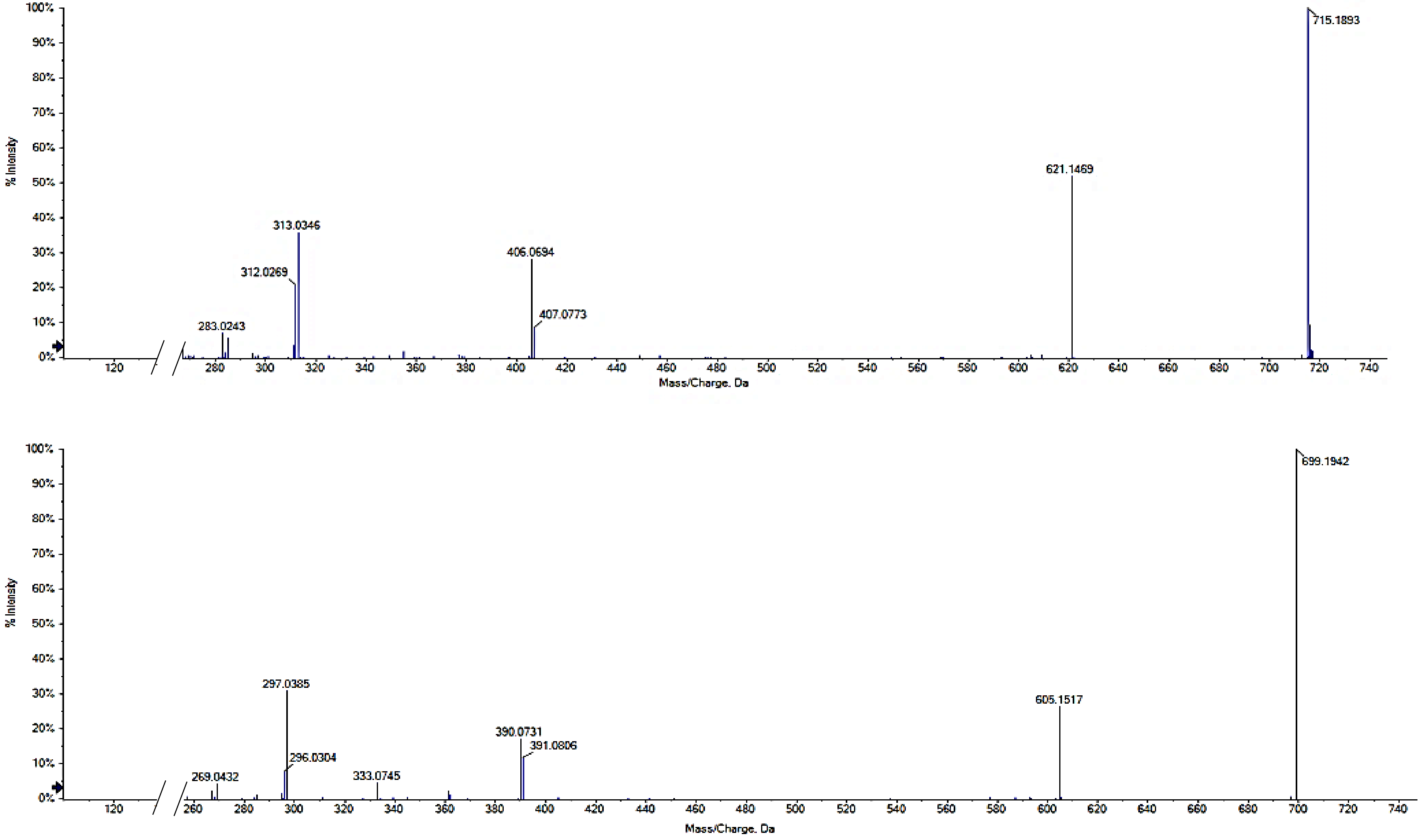

(b)

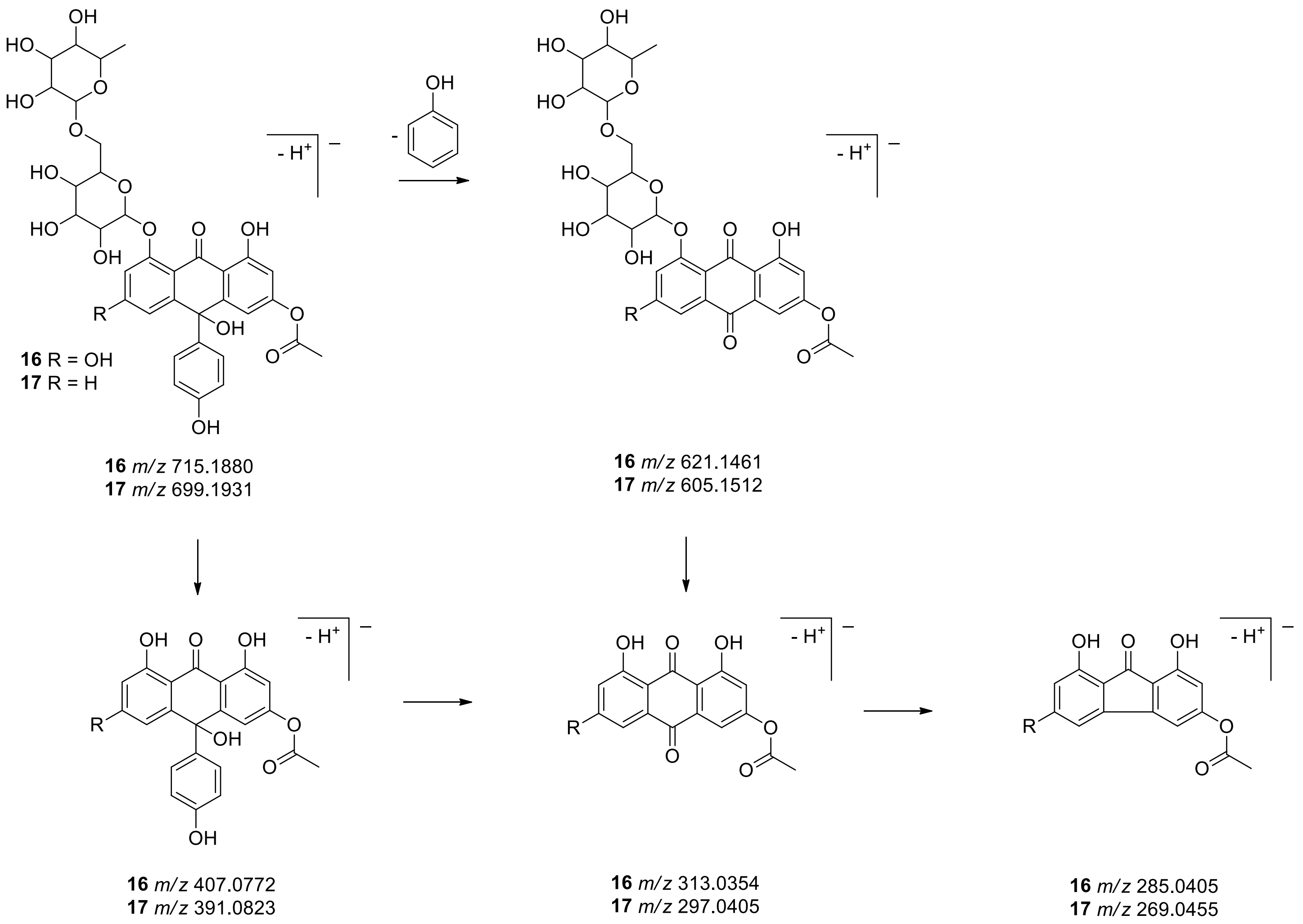

- H+
-
16 R = OH
17 R = H
16 m/z 715.1880
17 m/z 699.1931
16 m/z 621.1461
17 m/z 605.1512
16 m/z 407.0772
17 m/z 391.0823
16 m/z 313.0354
17 m/z 297.0405
16 m/z 285.0405
17 m/z 269.0455

Figure S3. TOF-MS spectra of (a) the ent-kaurene diterpene glycoside (compound **18**) tentatively identified in ZLF-A fraction; (c) rebaudioside A commercial standard. TOF-MS² spectra of (b) the ent-kaurene diterpene glycoside (compound **18**) tentatively identified in ZLF-A fraction; (d) rebaudioside A commercial standard.

(a)

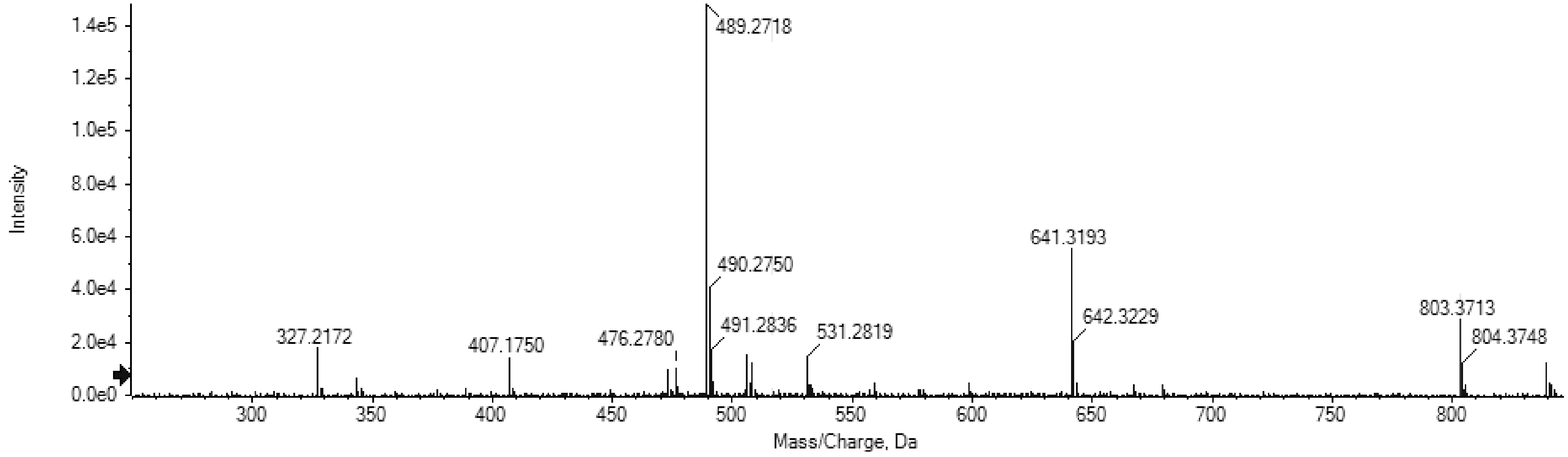

(b)

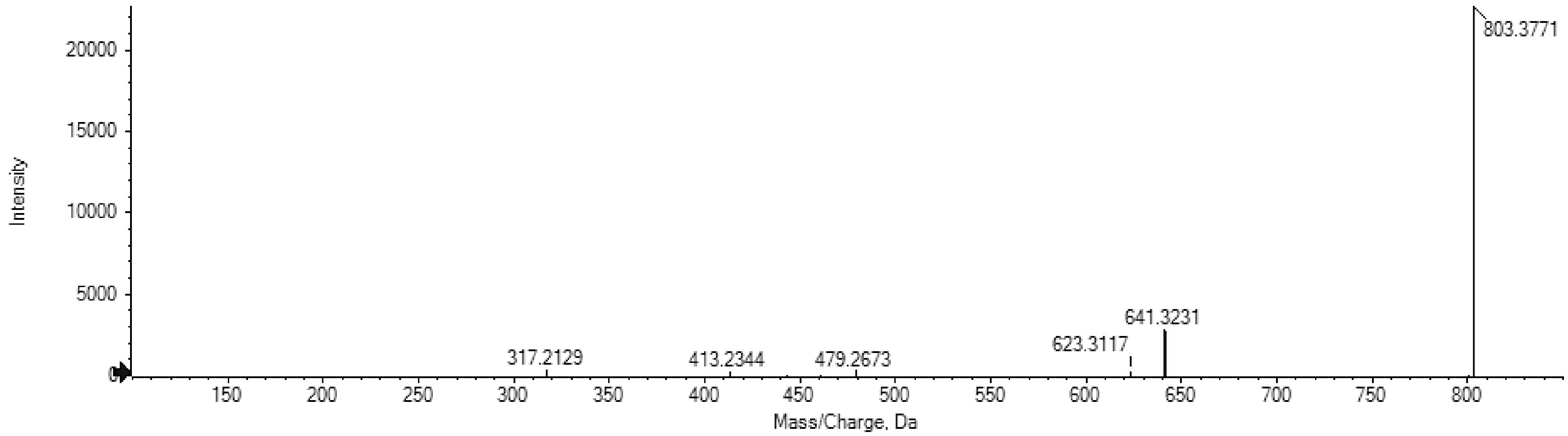

(c)

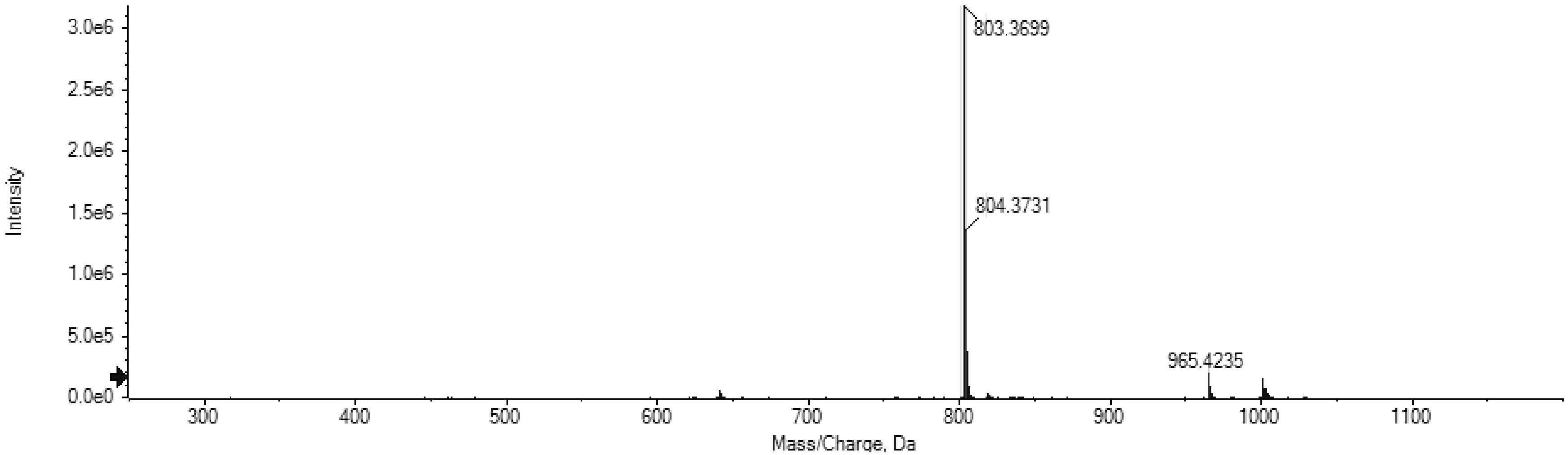
803.3699
804.3731
965.4235
Intensity
3.0e6
2.5e6
2.0e6
1.5e6
1.0e6
5.0e5
0.0e0
300
400
500
600
700
800
900
1000
1100
Mass/Charge, Da

(d)

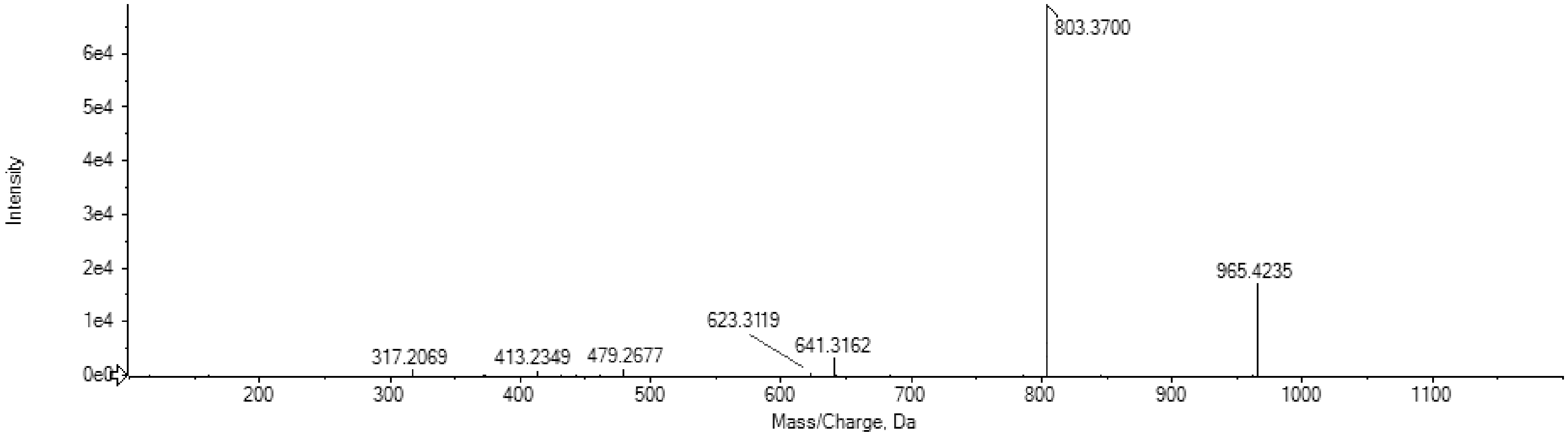
803.3700
965.4235
623.3119
641.3162
317.2069
413.2349
479.2677
Intensity
6e4
5e4
4e4
3e4
2e4
1e4
0e0
200
300
400
500
600
700
800
900
1000
1100
Mass/Charge, Da

Table S1. Quantitative data of ZLF-A identified constituents.

Peak n.	Rt (min)	Tentative assignment	Amount in ZLF-A fraction (μg mL^{-1})
**1**	8.80	Myricetin 3-*O*-hexoside	trace
**2**	9.33	Quercetin 3-*O*-dideoxyhexosyl-hexoside	20.8±2.2
**3**	9.71	Quercetin 3-*O*-hexosyl-pentoside	2.0±0.8
**4**	10.64	Kaempferol 3-*O*-(2'',6''-di-*O*-deoxyhexosyl)hexoside (e.g. clitorin)	46.3±6.2
**5**	10.72	Rutin	123.0±18.3
**6**	10.97	Quercetin 3-*O*-hexoside	4.0±0.5
**7**	10.98	Isorhamnetin 3-*O*-(2'',6''-di-*O*-deoxyhexosyl)hexoside (e.g. typhaneoside)	36.1±4.6
**8**	11.19	Luteolin hexoside	1.0±0.09
**9**	11.20	Luteolin hexosyl-deoxyhexoside	0.9±0.02
**10**	11.36	Kaempferol hexosyl-deoxyhexoside (isomer 1)	10.5±0.6
**11**	11.41	Kaempferol 3-*O*-hexosyl-pentoside (e.g. sambubioside)	1.5±0.09
**12**	11.65	Isorhamnetin 4′-*O*-rutinoside	7.3±0.1
**13**	12.27	Kaempferol hexosyl-deoxyhexoside (isomer 2)	96.7±14.2
**14**	12.27	Kaempferol 3-*O*-hexoside	trace
**15**	12.60	Isorhamnetin 7-*O*-rutinoside	62.2±8.7
**16**	13.31	Anthraquinone derivative 1	
**17**	13.53	Anthraquinone derivative 2	
**18**	13.73	Ent-kaurene diterpene glycoside	3.4±0.8

Rutin ($y=125837x+26331$; $R^2=0.9998$) was used as reference standard for identified glycosylated flavonoids and rebaudioside A ($y=208034x-1638.2$; $R^2=1$) for the ent-kaurene diterpene glycoside.
